# Case Report: A case of refractory tuberculous peritonitis mimicking and complicating suspected encapsulating peritoneal sclerosis in a long-term peritoneal dialysis patient

**DOI:** 10.3389/fmed.2026.1777805

**Published:** 2026-04-02

**Authors:** Tianjiao Cui, Mingcheng Huang

**Affiliations:** Department of Nephrology, Center of Kidney and Urology, The Seventh Affiliated Hospital, Sun Yat-sen University, Shenzhen, China

**Keywords:** differential diagnosis, encapsulating peritoneal sclerosis, metagenomic next-generation sequencing, peritoneal dialysis, tuberculous peritonitis

## Abstract

**Background:**

Tuberculous peritonitis (TBP) is a rare but severe complication in peritoneal dialysis (PD) patients, often presenting with non-specific symptoms. Its diagnosis is particularly challenging in patients with pre-existing or co-existing peritoneal pathology, such as changes suggestive of encapsulating peritoneal sclerosis (EPS).

**Case presentation:**

A 59-year-old male on PD for 14 years with no prior history of peritonitis presented with recurrent abdominal pain, fever, and cloudy effluent, following a recent episode of *Staphylococcus caprae* peritonitis. Initial contrast-enhanced computed tomography (CT) revealed diffuse peritoneal thickening, omental “caking,” and localized ascites, raising strong suspicion for EPS. However, the patient’s condition relapsed despite broad-spectrum antibiotic therapy. Metagenomic next-generation sequencing (mNGS) of peritoneal fluid definitively identified *Mycobacterium tuberculosis* complex. The diagnosis was thus revised to TBP manifesting with secondary peritoneal inflammatory changes mimicking EPS. Management involved laparoscopic PD catheter removal, transition to hemodialysis, and initiation of a renal-adjusted anti-tuberculous regimen (levofloxacin and linezolid), leading to gradual clinical and biochemical improvement.

**Conclusion:**

This case highlights that TBP can clinically and radiologically mimic EPS in long-term PD patients, leading to diagnostic delay. High clinical suspicion and the utilization of advanced molecular diagnostics like mNGS are crucial for accurate diagnosis. Catheter removal combined with appropriate anti-tuberculous therapy forms the cornerstone of management in such complex scenarios.

## Introduction

Peritoneal dialysis (PD)-associated peritonitis remains a major cause of morbidity and technique failure. While most cases are bacterial, tuberculous peritonitis (TBP) accounts for <2% of episodes but carries significant mortality if diagnosis is delayed (1, 2). TBP often presents insidiously with abdominal pain, cloudy effluent, and fever, closely mimicking bacterial peritonitis or other chronic peritoneal processes (3).

Encapsulating peritoneal sclerosis (EPS) is a devastating complication of long-term PD, characterized by progressive peritoneal fibrosis, bowel encapsulation, and obstructive symptoms (4). Its diagnosis relies on a combination of clinical symptoms (e.g., abdominal pain, vomiting, weight loss) and specific imaging findings on CT, such as peritoneal thickening, calcification, bowel tethering, and localized ascites (5).

The clinical and radiological presentations of TBP and EPS can overlap significantly. Both can cause peritoneal thickening, omental involvement, and ascites. This similarity poses a profound diagnostic challenge, as the treatment strategies are diametrically opposed: immunosuppression (e.g., corticosteroids) may be considered for EPS but is contraindicated in active tuberculosis (6, 7). We present a instructive case of a long-term PD patient in whom TBP initially masqueraded as EPS radiologically, and subsequently progressed to histopathologically confirmed EPS 15 months after successful anti-tuberculous therapy, underscoring the importance of definitive microbiological diagnosis and long-term surveillance.

## Case presentation

A 59-year-old man with end-stage renal disease (ESRD) secondary to chronic glomerulonephritis and hypertension had been on continuous ambulatory peritoneal dialysis (CAPD) for 14 years. Importantly, the patient had no prior history of peritonitis during these 14 years of PD. The current presentation represented his first-ever PD-related infection. He was admitted with a 6-day history of recurrent right-sided abdominal pain and fever. One month prior, he was hospitalized for bacterial peritonitis (*Staphylococcus caprae*) and was treated with a course of intraperitoneal antibiotics (piperacillin-tazobactam, meropenem, and vancomycin), with initial improvement. On current admission, he appeared chronically ill and cachectic. Vital signs were stable except for a low-grade fever. Abdominal examination revealed distension, diffuse tenderness, and cloudy PD effluent. Detailed examination revealed visible intestinal patterns without peristaltic waves, hyperactive bowel sounds, generalized tenderness with rebound, and no palpable masses. Murphy’s sign was negative. Digital rectal examination revealed no masses and no blood. Initial laboratory tests showed anemia (Hb 107 g/L), severe hypoalbuminemia (16.5 g/L), and markedly elevated inflammatory markers (C-reactive protein 143.28 mg/L, procalcitonin 2.47 ng/mL).

**Figure 1:**
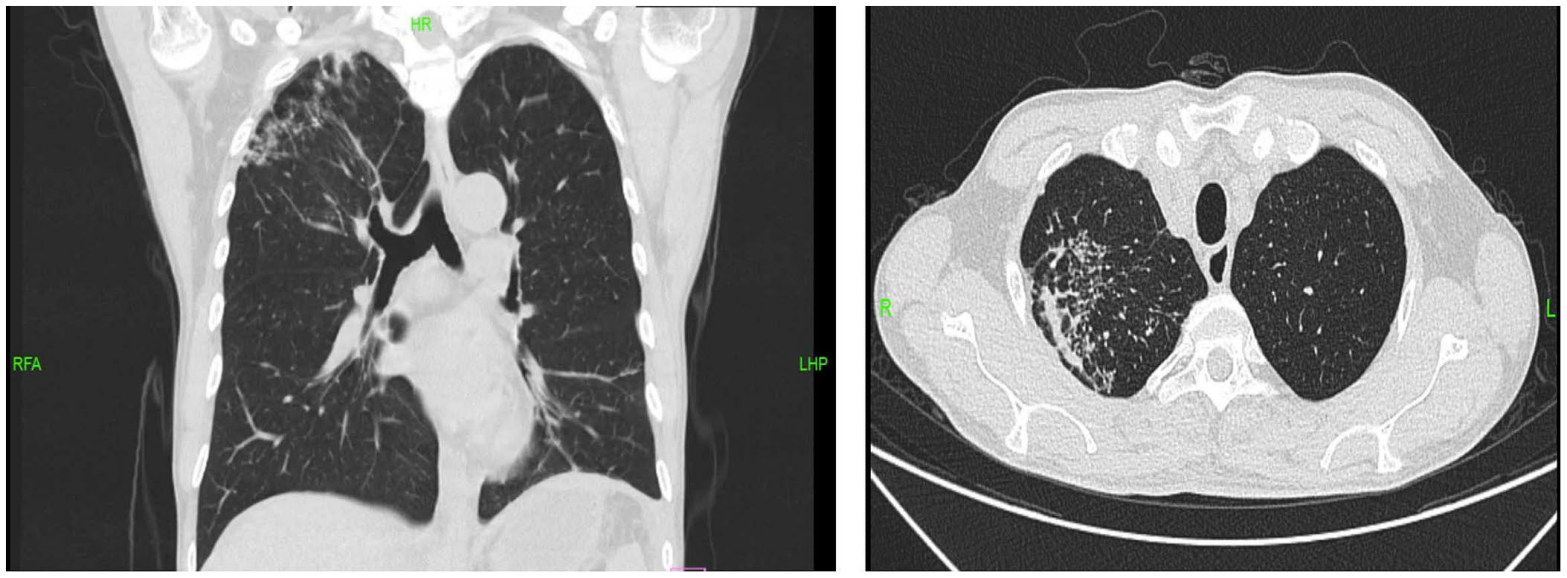
Chest CT shows multiple patchy and nodular opacities in the right upper and left lower lobes, with partial calcification and a tree-in-bud pattern adjacent to pleural thickening.

**Figure 2:**
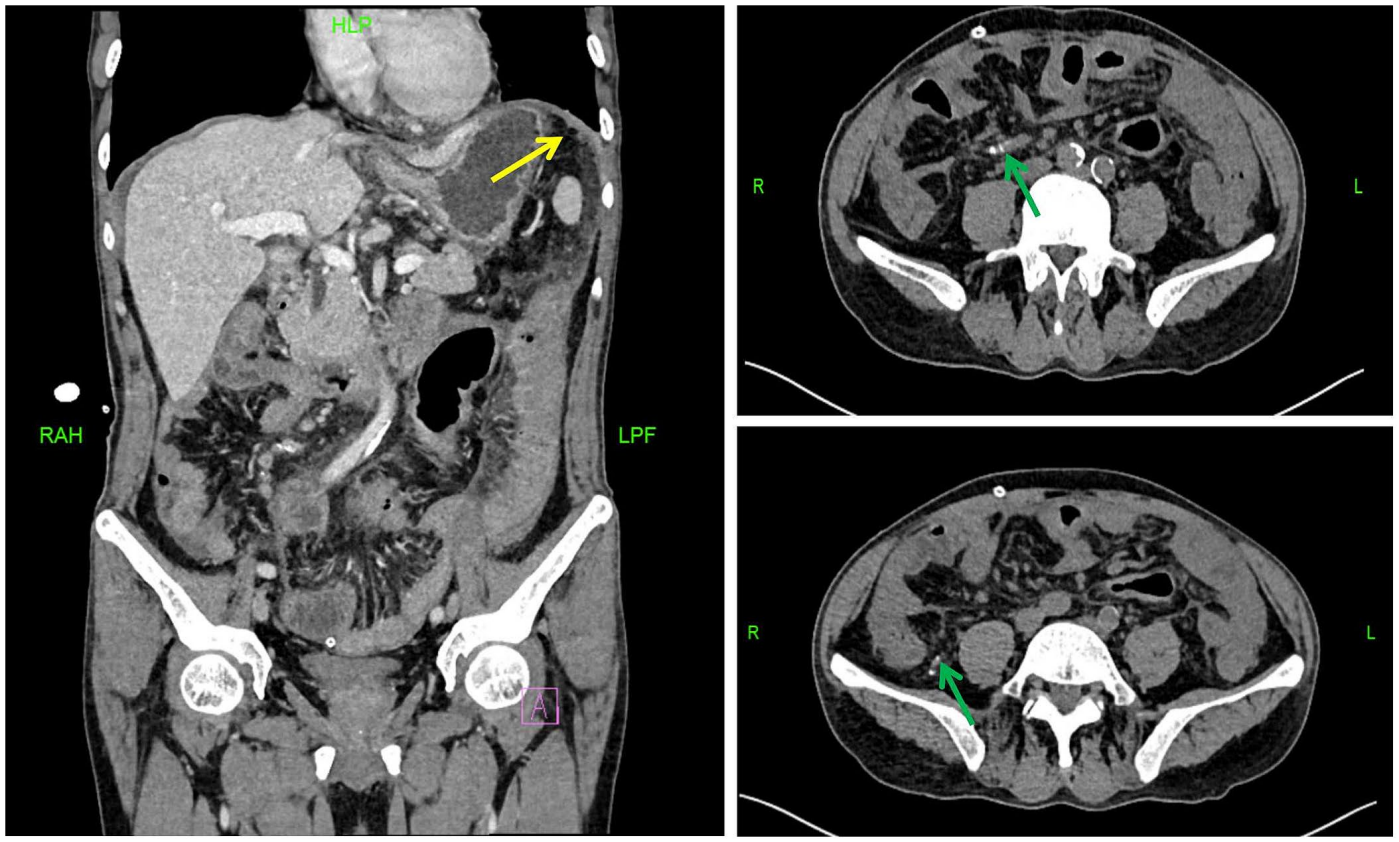
Contrast-enhanced abdominal CT shows thickening and blurring of the omentum around the stomach and transverse colon (yellow arrows), with partial omental calcification (green arrows).

A chest and abdominal CT scan with contrast was performed. The chest CT (Figure 1) showed old fibrotic and calcified lesions in the right upper lobe, suggestive of prior tuberculosis exposure. To exclude active pulmonary tuberculosis, three consecutive early-morning sputum specimens were obtained for AFB smear, mycobacterial culture, and TB-PCR; all were negative. Sputum testing for rifampicin resistance was also negative. The patient had no respiratory symptoms. Comparison of chest CT from October 2024 and January 2026 showed stable fibrotic lesions with no new cavitary changes, confirming inactive, old tuberculosis. The patient denied any known family history of tuberculosis or close contact with individuals with active TB. Abdominal CT (Figure 2) findings included diffuse smooth peritoneal thickening, omental infiltration and “caking,” mesenteric stranding, and localized ascites. These findings, in the context of long-term PD and recurrent peritonitis, were highly concerning for EPS, and this became a leading diagnostic consideration at this stage. Due to the relapse of symptoms and the gravity of a potential EPS diagnosis, a broad diagnostic workup was initiated. While standard cultures were pending, metagenomic next-generation sequencing (mNGS) was performed on the peritoneal fluid. mNGS of peritoneal fluid (probe capture method) revealed 5,479 sequence reads of *Mycobacterium tuberculosis* complex (relative abundance 66.15%, confidence 99%), establishing the diagnosis of tuberculous peritonitis. No pathogenic Gram-positive or Gram-negative bacteria were detected. Human herpesvirus 6B (HHV-6B, 2,247 reads) was also identified but considered clinically insignificant given normal immune status. This pivotal finding redirected the diagnosis from primary EPS to tuberculous peritonitis.

**Figure 3:**
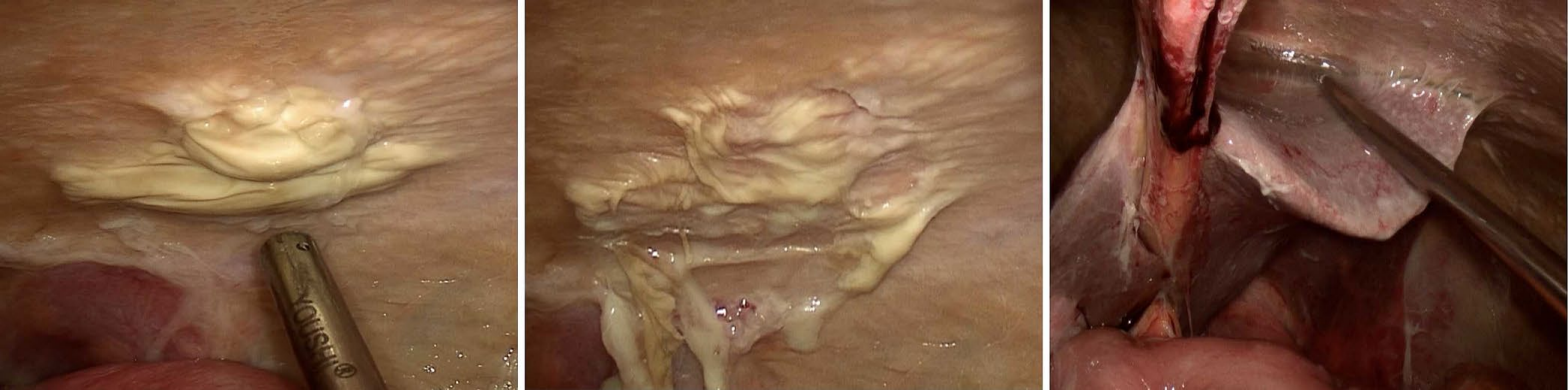
Intraoperative findings revealed extensive purulent exudate covering the parietal peritoneum with biofilm formation, as well as adhesions between the liver surface and segments of the intestine.

A multidisciplinary team was convened. Given the diagnosis of TBP and the PD catheter as a potential nidus of infection, laparoscopic PD catheter removal was performed. The intraoperative (Figure 3) findings included purulent exudate, fibrinous deposits, and adhesions, consistent with severe chronic inflammation but not the classic bowel encapsulation of advanced EPS. Anti-tuberculous therapy was initiated with a quad regimen (isoniazid 300 mg, rifampin 600 mg, pyrazinamide 1,000 mg, and ethambutol 800 mg daily), adjusted for renal failure. However, the patient developed severe drug-induced liver injury after 11 days, necessitating discontinuation. Following hepatoprotective therapy, a modified regimen of levofloxacin (500 mg daily) and linezolid (600 mg every 12 h) was successfully instituted and tolerated. This combination represents a non-standard salvage regimen necessitated by first-line drug intolerance and should not be interpreted as first-line therapy for tuberculous peritonitis. The patient completed a total of 6 months of anti-tuberculous therapy. Nutritional support with albumin infusions and enteral supplementation was aggressively pursued. The patient was transitioned permanently to hemodialysis via a newly created arteriovenous fistula.

After a prolonged hospitalization of 62 days, the patient was discharged. His abdominal pain resolved, inflammatory markers normalized, and nutritional status improved.

### Follow-up and outcomes

The patient completed 6 months of anti-tuberculous therapy by December 2024 and was transitioned to maintenance hemodialysis.

Initial Post-Treatment Period (January–September 2025): The patient remained clinically stable with resolved abdominal pain, normalized inflammatory markers (C-reactive protein, procalcitonin), and gradual improvement in nutritional status (albumin increased from 16.5 g/L at admission to 29.4 g/L by December 2024). Hemoglobin ranged from 60 to 88 g/L, managed with erythropoiesis-stimulating agents for renal anemia.

Recurrent Symptoms and EPS Progression (October 2025–January 2026): Beginning October 2025, the patient developed recurrent episodic abdominal pain, nausea, vomiting, and symptoms of incomplete intestinal obstruction requiring multiple hospitalizations. Abdominal CT in January 2026 revealed dilated small bowel loops with air-fluid levels (consistent with intestinal obstruction) and progressive peritoneal thickening with bowel tethering—findings highly suggestive of encapsulating peritoneal sclerosis (EPS).

Surgical intervention and pathological confirmation (January 22, 2026): Given persistent obstructive symptoms, the patient underwent exploratory laparotomy with complex pelvic adhesionlysis. Intraoperative findings revealed extensive fibrous adhesions and encapsulating membranous structures. Histopathological examination demonstrated fibroconnective tissue forming cyst wall-like structures, with focal lymphocytic and foam cell infiltration within the cyst wall, and homogeneous eosinophilic hyalinized collagenous material within the cysts—findings diagnostic of encapsulating peritoneal sclerosis. No malignant features were identified.

Postoperative course and final follow-up (January–March 2026): the patient recovered uneventfully with resolution of obstructive symptoms, return of bowel function, and resumption of regular hemodialysis.

At the most recent follow-up (March 2026), the patient remained clinically stable, ambulatory, and tolerating hemodialysis well with no recurrence of abdominal pain or obstructive symptoms. The total follow-up duration from TB diagnosis to the latest assessment is 17 months Table 1. Disease Timeline (2010–2026). Key events in the patient’s clinical course, from PD initiation to EPS diagnosis.

**Table 1:** A comprehensive timeline showing all key events from 2010 to 2026.

Date	Event	Key findings
2010	PD initiated	ESRD, CAPD started
2010–2024	14 years with NO peritonitis	Uncomplicated PD course
September 2024	First symptoms	Abdominal pain, cloudy effluent; likely early TB
October 2024	Hospitalization	CT: peritoneal thickening, omental caking (EPS suspected)
October 29, 2024	Catheter removal, mNGS	*M. tuberculosis* 5,479 reads – TB peritonitis diagnosed
November 2024	Anti-TB therapy	Quadruple regimen → hepatotoxicity → salvage regimen
December 2024	Treatment completed	6 months total therapy
October 2025	Symptoms recur	Abdominal pain, vomiting, obstruction
January 22, 2026	Surgery	Adhesion lysis, pathology confirms EPS
March 2026	Final follow-up	Clinically stable on hemodialysis

## Discussion

This case serves as a critical reminder of the diagnostic pitfalls in managing complex peritonitis in PD patients and offers several key insights: the initial CT findings in our patient were indistinguishable from those described in EPS (5, 8). The presence of long-term PD, recurrent peritonitis, and such radiological features naturally led to a strong suspicion of EPS. This case powerfully illustrates that TBP can produce secondary peritoneal inflammatory and fibrotic reactions that closely mimic the radiological signature of EPS. This overlap can lead to misdiagnosis, with potentially grave consequences if immunosuppressive therapy for presumed EPS is initiated in undiagnosed tuberculosis (7).

Importantly, this patient had no prior peritonitis over 14 years of PD; his first infection was TB peritonitis, which subsequently progressed to histopathologically confirmed EPS 15 months after successful anti-TB therapy. This suggests that TB infection alone may serve as a sufficient inflammatory trigger to initiate the fibrotic cascade leading to EPS. The relapse after bacterial peritonitis mandated an expanded differential diagnosis. While AFB smear and culture have low sensitivity and are slow, mNGS provided a rapid and definitive diagnosis, directly altering management (9). In this case, mNGS detected 5,479 *M. tuberculosis* reads while simultaneously excluding bacterial co-infection, demonstrating its particular value in paucibacillary tuberculous peritonitis where traditional methods have low sensitivity. Table 2 provides a comprehensive comparison of all diagnostic tests performed. Rather than presenting a definitive case of co-existing EPS and TBP, our patient’s course suggests that the radiological changes were most likely a manifestation of the intense granulomatous inflammatory response to *M. tuberculosis*. The chronicity of PD might have provided a predisposing peritoneal environment. This distinction is academically important as it shifts the primary pathophysiology from idiopathic progressive fibrosis to an infective-inflammatory cause with a potentially different prognosis and therapeutic focus. The subsequent development of EPS 15 months after successful TB treatment, confirmed by histopathology, suggests that TB infection may not only mimic EPS but also serve as a trigger for its development. The management underscores established principles for complicated PD-associated infections: source control (catheter removal) is paramount (1). The switch to hemodialysis was necessary due to the severely compromised peritoneal membrane. Tailoring anti-tuberculous therapy to account for renal impairment and close monitoring for adverse effects, particularly hepatotoxicity, is crucial. The successful use of a levofloxacin-linezolid salvage regimen after initial drug intolerance highlights the need for flexibility and expert pharmacologic management, though it must be emphasized that this represents a non-standard salvage regimen necessitated by first-line drug intolerance rather than first-line therapy.

**Table 2:** Comprehensive tuberculosis diagnostic testing results.

Test method	Specimen	Result	Turnaround time
AFB smear (3 consecutive)	Sputum	Negative	Hours
Mycobacterial culture	Sputum	No growth	Weeks
TB DNA PCR	Sputum	Negative	2 days
T-SPOT.TB	Whole blood	Positive	2 days
TB antibody	Serum	Positive	1 day
mNGS	Peritoneal fluid	*M. tuberculosis* 5,479 reads	2 days
Histopathology	Peritoneal tissue	EPS diagnosed	5 days

Several limitations of this case report should be acknowledged. As a single case report, the findings may not be generalizable to all patients with tuberculous peritonitis. While the temporal association between TB infection and subsequent EPS development is compelling, causality cannot be definitively established from a single observation. The anti-tuberculous regimen used was non-standard, as linezolid-levofloxacin represents salvage therapy necessitated by first-line drug intolerance. The clinical significance of the detected HHV-6B co-infection remains uncertain. Additionally, the absence of pre-TB peritoneal biopsy means that subclinical peritoneal changes predisposing to EPS cannot be entirely excluded. The surgical intervention for obstructive symptoms precluded observation of the natural progression of post-TB EPS. Finally, although the patient has been followed for 17 months, this duration may not capture very late EPS progression.

## Conclusion

Tuberculous peritonitis should be considered a great mimicker in long-term PD patients, capable of presenting with clinical and radiological features indistinguishable from encapsulating peritoneal sclerosis. Clinicians must maintain a high index of suspicion in cases of relapsing or refractory peritonitis. The integration of advanced molecular diagnostics like mNGS into the diagnostic pathway is essential to avoid misdiagnosis and guide appropriate, potentially life-saving therapy. The cornerstone of management involves a combination of timely catheter removal, initiation of carefully monitored anti-tuberculous chemotherapy, and robust supportive care.

## Data Availability

The original contributions presented in the study are included in the article/supplementary material, further inquiries can be directed to the corresponding author.
